# Towards host-directed therapy: The role of matrix metalloproteinases in the immunopathogenesis of tuberculosis

**DOI:** 10.1016/j.ijid.2026.108525

**Published:** 2026-06

**Authors:** Maria-Cristina I. Loader, Deborah L.W. Chong, Keira H. Skolimowska, Jon S. Friedland

**Affiliations:** Institute for Infection and Immunity, School of Health and Medical Sciences, City St George’s, University of London, London, UK

**Keywords:** Tuberculosis, Immunopathology, Matrix metalloproteinases, Host-directed therapies

## Abstract

Matrix metalloproteinases (MMPs) are zinc-dependent proteases that sit at the interface of inflammation, tissue injury, and repair in tuberculosis. In pulmonary tuberculosis, dysregulated MMP activity drives extracellular matrix degradation, cavitation, and remodelling that contribute to post-tuberculosis lung disease. In tuberculous meningitis, MMPs have been implicated in blood–brain barrier disruption and neuroinflammatory injury. Experimental and clinical research consistently link elevated MMPs with radiological damage and adverse outcomes, while mechanistic studies highlight the importance of cellular networks in amplifying matrix-destructive pathways. MMP activity is further shaped by host modifiers, such as co-infection, and the pathological microenvironment characterised by metabolic stress, acidosis and hypoxia. Host-directed therapies are interventions that target dysregulated host immune and tissue injury pathways, rather than *Mycobacterium tuberculosis* itself, with the aim of limiting pathology and improving clinical outcomes. Preclinical evidence supports modulation of matrix degradation, exemplified by doxycycline-mediated suppression of pathological MMP activity and improved survival in selected pulmonary and central nervous system TB models. Future progress will depend on translating these insights into clinical benefit with carefully designed studies that consider host heterogeneity, co-infection, and disease compartment to refine matrix-targeted host-directed approaches in an era of increasingly personalised medicine.

## Highlights

• Matrix metalloproteinases drive tissue destruction in TB.
• MMPs drive inflammation, cavitation and long-term sequelae.
• Co-infection and lesion physiology shape MMP activity.
• MMP-targeted HDTs show proof-of-concept in early trials.
• Precision strategies are needed to limit TB immunopathology.

## Introduction

Despite effective antimicrobial therapy, tuberculosis (TB) continues to present substantial challenges worldwide, including increasing resistance to new drugs, prolonged treatment time and chronic tissue damage. Host inflammatory injury and tissue destruction are major contributors to death from active TB. Among those who survive infection, long-term all-cause mortality remains substantially higher than in the general population, reaching up to six-fold in some cohorts [[1], [2], [3]]. Post-tuberculosis lung disease (PTLD) encompasses a spectrum of chronic respiratory sequelae following microbiological cure of pulmonary TB (PTB), including airflow obstruction, restrictive ventilatory defects, impaired gas exchange, and structural abnormalities such as fibrosis and cavitation, which together contribute to persistent symptoms, functional limitation, and increased risk of respiratory mortality [3,4]. Tuberculous meningitis (TBM) is associated with the highest mortality of all forms of TB, and long-term neurological disability is common among survivors, reflecting severe inflammatory injury and disruption of the blood–brain barrier (BBB) within the central nervous system (CNS) [5].

Current TB treatment strategies focus predominantly on pathogen eradication, with limited attention to the host immune responses that drive tissue damage and may influence tissue penetration of anti-mycobacterial drugs. In PTB, this dysregulation contributes to the lung tissue destruction and pathological remodelling that underpin PTLD. In TBM, immune-mediated injury drives BBB disruption and downstream complications including basal meningeal exudates, hydrocephalus and cerebral infarction. Host-directed therapies (HDTs) are adjunctive interventions that do not target the bacterium, *Mycobacterium tuberculosis* (*Mtb*), directly but instead aim to accelerate disease resolution, enhance antimicrobial activity, and/or mitigate the effects of the innate inflammatory response to infection [6,7].

Several HDTs have been evaluated as adjuncts to standard treatment for PTB, predominantly in early-phase clinical trials in humans. These studies have largely involved repurposed agents with immunomodulatory or metabolic effects, including corticosteroids, vitamin D, metformin and statins [8]. Trial designs have primarily focused on safety, biological activity, and intermediate outcomes, such as immunological markers, inflammatory pathways, or sputum culture conversion, rather than being powered for hard clinical endpoints such as mortality, lung function, or long-term disability. While some interventions have demonstrated measurable biological or immunological effects, evidence for consistent and clinically meaningful benefit has been limited and heterogeneous across studies. No adjunctive HDT has shown sufficient, reproducible efficacy to support routine clinical use. In TBM, dexamethasone remains the only evidence-based adjunctive HDT, conferring a modest reduction in short-term mortality but no improvement in long-term neurological disability among survivors [9,10]. Collectively, these studies highlight the limitations of broadly acting immunomodulatory strategies in TB and underscore the need for host-directed interventions that are mechanistically informed and evaluated against clinically meaningful endpoints.

One promising approach within the HDT framework is to target the mechanisms underpinning extracellular matrix (ECM) degradation. Matrix metalloproteinases (MMPs), a family of zinc-dependent proteolytic enzymes, play a central role in tissue destruction in TB. In PTB, MMPs drive ECM degradation, cavitation, and pathological remodelling, while in TBM they have been implicated in BBB breakdown and neuroinflammatory injury. These enzymes are important effectors of immune-mediated tissue damage and have been extensively characterised in experimental, translational, and clinical studies in TB [4,[11], [12], [13]]. Their position at the interface between inflammation, tissue injury, and repair make MMPs attractive targets for therapeutic intervention aimed at limiting acute tissue damage and reducing long-term post-TB sequelae.

This review focuses on regulating MMPs as potential HDTs in TB, exploring their mechanistic role in disease and their potential to reshape TB treatment paradigms beyond pathogen eradication alone. We first summarise pulmonary and CNS TB immunopathology, then discuss host modifiers of MMP activity, before reviewing therapeutic approaches and proposing future research priorities.

## Tuberculosis immunopathology

### Pulmonary TB

Granuloma formation is central to TB pathology, acting to contain the infection but also providing a niche for *Mtb* persistence. Granulomas are structured lesions with monocyte/macrophage-derived cells at the core, surrounded by T cells and stromal cells, such as fibroblasts, which coordinate immune responses defining disease progression. Granulomas may undergo central caseous necrosis and subsequently *Mtb*-induced chronic inflammation and tissue remodelling may result in deposition of fibrotic tissue, leading to significant and often irreversible impairment of lung function [4].

PTB progression is marked by cavitation, a destructive tissue process mediated by MMPs. The activity of MMPs is regulated at the transcriptional level and by secreted tissue inhibitors of MMPs (TIMPs). Animal studies have demonstrated the central role of MMP-1, a collagenase, in driving TB-induced tissue remodelling and cavitation [11]. Additionally, MMP-1, -2, -7, and -9 are significantly elevated in plasma from PTB patients relative to controls, with increased MMP expression independently correlating with poorer clinical outcomes [12], supporting the role of MMPs in driving PTB immunopathology.

Cellular sources of MMPs during direct *Mtb* infection include innate immune cells. We have previously shown that *Mtb*-infected monocyte-derived macrophages express MMP-1, -3, -7, and -10 [14]. Neutrophils, one of the predominantly *Mtb*-infected cell populations in the airways of TB patients, can also directly secrete MMP-8 and -9. Moreover, *Mtb* infection of neutrophils induces release of neutrophil extracellular traps (NETs), consisting of extruded extracellular DNA scaffolds, interlaced with citrullinated histones and enzymes, including MMP-8, that promotes ECM breakdown as observed in TB patients [15].

Immune cell crosstalk occurs during *Mtb* infection to drive immunopathology. Cellular networks can activate lung epithelial cells and fibroblasts, amplifying matrix destruction via modulation of PI3K/AKT/mTOR- or p38 MAPK-dependent cell signalling pathways to upregulate expression of both pro-inflammatory cytokines and MMPs [16]. Additionally, platelets are increasingly recognised to possess immunomodulatory functions, alongside their role in coagulation. We found that platelet activation markers are significantly increased in the plasma of patients with TB, and that platelets enhance MMP-1 and -10 secretion from monocytes during *Mtb* infection [17]. Overall, these studies highlight the importance of *Mtb*-cellular networks that contribute towards an MMP-dependent matrix-degrading phenotype, to drive tissue damage [18].

### TB meningitis

TBM, the most severe manifestation of TB, occurs following haematogenous dissemination of *Mtb* to the CNS, crossing the blood-cerebrospinal fluid (CSF) and BBB [5,13]. MMPs have been implicated in BBB breakdown in TBM and sources of MMPs in the CNS are likely a combination of infiltrating leukocytes as well as CNS-resident cells, including microglia and astrocytes. The gelatinases, MMP-2 and -9, which degrade type IV collagen, a key structural component of the BBB, have been most extensively studied. Although elevated CSF concentrations of MMP-2 and -9 are consistently reported in TBM [18,19], and there is compelling evidence that MMP-9 is integral to BBB breakdown [20], associations with clinical outcome have been inconsistent.

Broader profiling studies implicate additional MMPs in determining TBM outcome. CSF MMP-7 and -10 have been associated with severe disability and mortality in CNS TB [21,22]. MMP-10 can activate other MMPs (MMP-1, -8, -9) and is thought to be functionally important in TBM-associated tissue damage [23]. MMP-3, a stromelysin, and the collagenase MMP-1, are highly expressed in CNS granulomas, suggesting they contribute to tissue destruction at the site of disease [24].

Our group demonstrated that dexamethasone was associated with reduced CSF concentrations of neutrophil-derived MMP-8 and MMP-9, offering mechanistic insight into its protective effect [25]. Although neutrophil-derived MMP-8 has been relatively under-studied in TBM, emerging evidence implicates neutrophils as drivers of CNS immunopathology [26]. NETs are present in CNS granulomata and CSF. They can promote thrombosis, a major complication of TBM, and are associated with poor clinical outcomes [21]. Peripheral MMP-8 expression has recently been linked to cerebral infarction in paediatric TBM [27]. Together, these observations implicate MMPs, and in particular neutrophil-associated protease pathways, as central mediators of BBB disruption and tissue injury in TBM.

## Modifiers of MMP biology and tissue destruction in TB

There are many factors that potentially modify MMP activity in TB but two of the most important are co-infection with a second pathogen and pathophysiological changes within the host. Although multiple pathogens can coexist with *Mtb*, this section focuses on HIV and soil-transmitted helminths (STHs), two globally prevalent co-infections with distinct but convergent effects on host inflammatory regulation. These co-infections provide complementary models for understanding how external biological pressures reshape MMP biology and tissue injury in TB.

### Co-infection

#### HIV-TB co-infection

HIV co-infection is the strongest risk factor for progression from *Mtb* infection to active disease and is independently associated with unfavourable treatment outcomes. Consequently, TB remains the leading cause of death worldwide among people living with HIV.

Patients with advanced HIV and PTB rarely develop cavitary lung disease and have reduced sputum MMP-1 concentrations compared with HIV-negative TB patients, supporting a role for MMP-1 in matrix destruction in PTB [28]. HIV-TB coinfection is associated with more severe disease and poorer outcomes. Elevated plasma MMP-8, particularly in association with *Mtb* bloodstream infection, has been linked to mortality [29], linking MMP dysregulation with pathogenesis and adverse outcomes of HIV-associated TB.

Antiretroviral therapy (ART) improves survival in advanced HIV-TB but increases the risk of immune reconstitution inflammatory syndrome (TB-IRIS), a pro-inflammatory condition characterised by tissue damage. *In vitro, Mtb*-stimulated peripheral blood mononuclear cells from patients with TB-IRIS secrete higher levels of MMP-1, -3, -7 and -10 than those from non-IRIS controls [30]. Clinically, in a prospective cohort of HIV-associated PTB, ART-associated increases in plasma MMP-8 independently predicted TB-IRIS and were associated with reduced post-treatment lung function, implicating MMP dysregulation in both acute immunopathology and longer-term pulmonary impairment [31].

In HIV-associated TBM-IRIS, CSF neutrophil activation and elevated MMP concentrations persist despite adjunctive corticosteroid therapy [23], suggesting inadequate control of inflammation. Consistent with this, a recent randomised trial showed no mortality benefit of corticosteroids in HIV-associated TBM [10], underscoring the need for more targeted host-directed therapies to limit tissue damage in HIV-associated TB.

### Soil-transmitted helminth co-infection

STHs are among the most prevalent chronic infections worldwide, affecting an estimated 1.5 billion people as of 2021 [32,33], with the highest prevalence in regions that also carry a substantial incidence of TB [34]. In many TB-endemic settings across sub-Saharan Africa, South and Southeast Asia, and parts of Latin America, chronic helminth infection is therefore not an exception but a common immunological background state. Epidemiological and clinical studies link helminth co-infection to impaired anti-mycobacterial immune responses and adverse clinical outcomes during TB treatment [[35], [36], [37]], supporting a role for helminths as modifiers of TB susceptibility and disease course.

In clinical studies of PTB, helminth co-infection correlates with more severe radiological disease, greater lung destruction, and elevated circulating collagenases and neutrophil-associated MMPs, including MMP-1, -8, -9, and -10, implicating enhanced matrix degradation as an underlying mechanism [38,39]. Type 2 immune features commonly associated with helminth infection, including IgE production and eosinophil activation, may contribute by influencing macrophage activation states and MMP networks within the lung. Our recent data linking IgE-associated immune responses to increased MMP activity provide a mechanistic framework connecting helminth co-infection to MMP-driven lung damage in TB [40]. In this context, helminth infection represents a biologically plausible amplifier of matrix-destructive pathways rather than a coincidental co-morbidity.

Data on helminth co-infection in TBM are sparse. A prospective study of adults with TBM reported that *Strongyloides stercoralis* co-infection was associated with altered baseline CSF immune profiles, including lower concentrations of pro-inflammatory cytokines [41]. While this suggests helminths may modulate intracerebral inflammatory responses, evidence linking helminth co-infection to MMP activity or structural CNS injury in TBM remains limited.

These observations have direct implications for HDT development. In PTB, helminth co-infection may amplify immune-mediated lung injury through MMP-driven matrix degradation, identifying a defined subgroup in whom MMP-targeted adjunctive therapy may have enhanced impact. In co-endemic settings, failure to account for helminth-driven immune conditioning risks obscuring therapeutic effects in HDT trials and undermining precision approaches to host-directed intervention.

### Pathophysiological changes within TB lesions

Local pathophysiological states within TB patients potentially profoundly modify MMP activity and tissue destruction. Metabolic reprogramming influences MMP expression and tissue destruction during TB. Weight loss predisposes to TB [42] while diabetes mellitus represents a major risk factor for active disease and progression from latent infection [43]. ^18^F-FDG-PET imaging of active TB reveals elevated glucose uptake within lesions [44], consistent with increased metabolic activity and acute inflammation at the site of disease. At the cellular level, *Mtb*-infected macrophages undergo a shift from oxidative phosphorylation towards aerobic glycolysis, defined as the Warburg effect. This process shapes downstream inflammatory responses, including increased TNFα and IL-1β expression. Importantly, metabolic signalling pathways also directly regulate tissue remodelling, and work from our group has shown that neutrophil-derived MMP-8 expression during *Mtb* infection is dependent on AMPK, a signalling molecule that activates catabolic pathways [15]. Thus, metabolic reprogramming regulates both inflammatory and tissue remodelling processes in TB.

Localised tissue acidosis is a critical microenvironmental feature of TB lesions that modifies MMP expression and tissue remodelling and is closely linked to metabolic reprogramming [45]. Increased glycolytic activity in immune cells, including *Mtb*-infected macrophages, leads to lactate accumulation, and excessive lactate export with limited clearance generates an acidic lesion microenvironment [46]. Extracellular acidosis in the context of *Mtb* infection amplifies *Mtb*-induced IL-1β, MMP-1 and -3 secretion, while downregulating TNFα and IL-6 production [47]. Thus, acidosis biases the inflammatory milieu towards enhanced matrix degradation within TB lesions.

Lastly hypoxia, demonstrated by increased ^18^F-MISO PET tracer uptake within TB lesions [48], is another defining microenvironmental feature of TB lesions that modifies MMP expression and tissue remodelling. Hypoxia-inducible factor-1α (HIF-1α), a low oxygen sensor and transcription factor, is stabilised by hypoxic conditions and enhances the anti-microbial function of *Mtb*-infected macrophages while modulating inflammatory cytokine production, including reduced TNFα and IL-10 expression [49]. Furthermore, hypoxic signalling promotes tissue destruction, since HIF-1α stabilisation upregulates MMP expression in *Mtb*-infected innate immune cells [48,50]. In parallel, HIF-1α can promote glycolytic reprogramming by inducing enzymes such as lactate dehydrogenase A, diverting pyruvate towards lactate production. This amplifies a feed-forward loop that increases matrix degradation and tissue remodelling in TB [49].

In summary, tissue pathology in TB is shaped by coordinated host responses within infected lesions. Metabolic reprogramming and local microenvironmental changes are tightly interconnected and modulate inflammatory signalling and tissue remodelling through MMP activity. Understanding these processes is essential for the development of HDTs that not only target *Mtb* but also limit immune-mediated tissue damage.

## Targeting MMPs for HDT in TB

Accumulating experimental and clinical evidence indicates that MMPs, as key mediators of tissue destruction in TB, represent tractable targets for HDT. Preclinical studies have highlighted the therapeutic value of targeting MMPs with small molecule inhibitors that can be used in combination with standard anti-mycobacterial antibiotics, isoniazid and rifampicin. The use of marimastat, a broad-spectrum MMP inhibitor, reduced immune cell clustering and intracellular *Mtb* bacterial load in a cellular model of TB granuloma formation [51]. Furthermore, administration of marimastat in a murine model of TB led to stabilised lung tissue architecture and improved anti-TB drug delivery to infected sites [52]. Global MMP inhibition may both mitigate TB immunopathology and potentiate antimicrobial therapy.

Clinical evidence for MMP-targeted HDT, although still limited, is emerging. Doxycycline, an FDA-approved tetracycline antibiotic with broad-spectrum but weak MMP-inhibitory activity, showed promising results in a randomised, placebo-controlled phase II trial in patients with PTB [53]. In this study, a short course of adjunctive doxycycline suppressed pathological MMP activity, including MMP-1, −8, −9, −12 and −13, and reduced collagen and elastin degradation. Improvements in lung volume capacity were observed, alongside a statistically significant reduction in pulmonary cavity volume, from 23,694 mm³ at baseline to 1136 mm³ at week 8, representing an approximately 95 percent decrease in mean cavity size in the doxycycline arm. No significant reduction occurred in the placebo group (adjusted *P* = 0.045). Evidence supporting MMP-targeting also extends to CNS disease. In a murine model of TBM, adjunctive broad- and narrow-spectrum MMP inhibition improved survival [21], and a phase II human trial is currently investigating adjunctive doxycycline as HDT in TBM (NCT06446245). Together, these findings provide proof-of-concept that host-directed modulation of matrix degradation is feasible in humans, directly linking mechanistic understanding of tissue destruction to an actionable therapeutic strategy across pulmonary and CNS TB disease.

**Figure 1:**
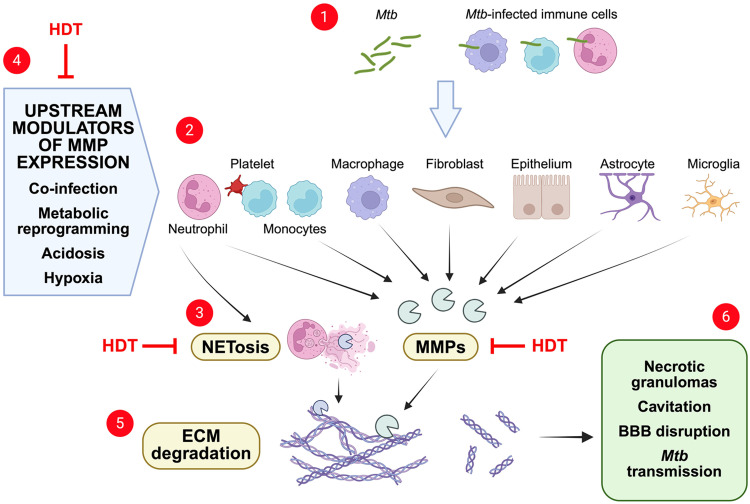
Potential host-directed therapeutic targets (HDT) to limit matrix metalloproteinase (MMP) function in tuberculosis (TB). After *Mycobacterium tuberculosis* (*Mtb)* bacilli enter the tissue, direct infection or cellular-*Mtb* networks (1) activate immune cells (e.g., neutrophils, monocytes, macrophages, astrocytes and microglia) or structural cells (e.g., fibroblasts or epithelial cells) to express MMPs (2). In addition, *Mtb* infection of neutrophils induces neutrophil extracellular traps (NETs), which contain MMPs, via a process called NETosis (3). Co-infection (e.g., soil-transmitted helminths) or other physiological responses, such as metabolic reprogramming, localised tissue acidosis or hypoxia may enhance expression of MMPs during *Mtb* infection (4). Expressed MMPs degrade extracellular matrix (ECM) components, including collagen and gelatin (5), to ultimately drive tissue destruction and *Mtb* transmission (6). Potential host-directed therapeutic targets to limit MMP activity include direct inhibition of MMP function (e.g., doxycycline), modulators of MMP expression (e.g., inhibition of phosphodiesterase or HIF-1α-dependent signalling) or modulators of NETosis (e.g., Dornase alfa).

Despite promising early preclinical and clinical data supporting MMP targeting as a host-directed strategy in TB (Figure 1), substantial challenges remain. This reflects the complexity of MMP biology, which may differ between groups of patients and the relative lack of specific MMP inhibitors. Early broad-spectrum MMP inhibitors have failed to translate clinically, largely due to unacceptable off-target toxicity such as dose-limiting musculoskeletal adverse effects [54,55]. Different MMPs have overlapping and context-dependent roles that contribute to both beneficial immune responses, such as cell migration and activation of inflammatory responses, and also immunopathology, such as tissue destruction and cavitation. This raises concerns that global inhibition of their harmful functions may also compromise beneficial host responses and decrease bacterial clearance.

Emerging experimental approaches aim to address these challenges through increased selectivity or indirect modulation of specific MMP activity, rather than global protease inhibition. Mechanistic studies indicate that adjunctive immunomodulatory strategies, including phosphodiesterase inhibition, can downregulate pathological MMP expression without directly targeting individual proteases [56]. In parallel, targeting upstream regulators of tissue remodelling, such as NETs with Dornase alfa, blocking acidosis-sensing receptors, or HIF-1α-dependent signalling pathways, offers alternative approaches to preventing matrix degradation while preserving essential immune functions [47,48,50,57,58]. It is clear that targeting host defence mechanisms independent of considering the different changes in physiology found in different patient groups is unlikely to be successful.

## Future perspective on HDT in TB

Advances in human tissue studies, longitudinal cohorts, and mechanistic ex vivo work have established MMPs as central effectors of tissue destruction in TB. Elevated activity of collagenases and gelatinases consistently associates with acute and chronic tissue damage, reframing protease-mediated matrix degradation as a modifiable component of TB pathogenesis rather than an unavoidable consequence of infection [15,38,39].

This shift in understanding has accelerated the use of MMPs and related markers of ECM turnover as biologically informative readouts in cohort studies and early-phase HDT trials. Longitudinal measurements of sputum and plasma MMPs, alongside markers of neutrophil activation and ECM turnover, capture tissue pathology that is poorly reflected by sputum bacillary burden alone [15,59]. In TBM, CSF MMPs and related inflammatory markers offer complementary insight into BBB integrity and intracerebral injury [20].

Development of MMP-targeted HDTs remains constrained by incomplete understanding of in vivo pharmacodynamics, uncertainty around optimal timing and tissue and patient specificity, as well as recognised safety and feasibility challenges highlighted by prior experience with broad-spectrum MMP inhibition [55,60]. Therapeutic strategies should move beyond broad-spectrum MMP inhibition towards more selective or context-dependent approaches, including targeting specific proteases, upstream regulatory pathways, or cell-specific sources of pathological MMP activity. Second, biomarker-informed adjunctive trials are needed to define optimal timing, dose, and duration of MMP modulation, taking into account both pre-existing physiological changes in patients such as found with low body mass index, as well as those occurring during active TB. Third, there is a strong rationale to extend MMP-targeted strategies beyond acute disease into areas such as PTLD, where ongoing matrix remodelling may remain amenable to intervention after microbiological cure. Collectively, these priorities define a practical research agenda for the next generation of MMP-focused HDTs, centred on trial design, patient stratification, and therapeutic specificity rather than proof-of-concept alone. However, it will be important to acknowledge that different patient sub-groups may respond to specific HDTs to varying extents.

## Conclusion

Targeting MMPs in TB is an attractive and feasible path for HDTs to complement anti-mycobacterial drug therapies. MMPs have key roles in tissue damage in both pulmonary and CNS TB both acutely and by causing chronic fibrosis. The evidence shows that although the immune response is complex and organ specific, MMPs do represent a common effector leading to patient morbidity and mortality. However, the principal issue is that the human immune system does not function in a vacuum but in the context of frequent or serious co-infections (for example with parasites and HIV respectively) and in the presence of both pre-existing and infection-stimulated physiological changes. Such factors will have marked influences on immune responses and are likely to vary significantly between groups of TB patients.

TB therapy has largely until now adopted a one-regimen-fits-all approach, which is unlikely to be sufficient for complex or drug-resistant disease, where HDTs are most urgently required. The future will almost certainly require a more personalised medicine approach to TB management, potentially at the level of individual patients, but more realistically across groups defined by shared disease modifiers and physiological changes. Understanding how these factors shape immune responses will be essential for the pre-clinical development of new host-directed therapies.

## Declaration of competing interest

The authors declare that they have no known competing financial interests or personal relationships that could have appeared to influence the work reported in this paper.
